# The Great Yellow Peril

**Published:** 1916-08

**Authors:** Charles H. Duncan

**Affiliations:** New York


### The Great Yellow Peril.

BY CHARLES H. DUNCAN, M. D., NEW YORK.

It is important in these perilous war times that we be "medically prepared" to meet the "Great Yellow Peril"—pus infection—by modern improved methods.

It is especially important that prejudice and bias should be cast aside by those who are entrusted with the lives of our soldiers; that we keep in step with the progress of the times, and profit by the experience taught us by the surgeons of the European armies; but we do not have to look to the surgeons of Europe, as autotheraphy is distinctly an American production and is vouched for by the highest medical and veterinary authorities in our own country.

Autotherapy is *the* treatment and the only treatment that will cure many severe and obstinate infections.

J. C. Parham, Past Assistant Surgeon of the United States Navy, states of autotherapy in the *Southern Medical Journal* of April, 1916, that "the day of antiseptics or germicides such as bichloride, phenol, etc., so generally employed in surgical practice, is on the wane, and the almost universal use of salt solutions, or salt solution and sodium citrate, will supplant them where the employment of autotherapy is anticipated."

F. W. Sumner, M. D., of Saharanpore, India, says in an article on autotheraphy under the title of "Prevention and Treatment of Septic Wounds in Warfare" that appeared in the *Indian Medical Gazette,* November, 1914: "The autotherapeutic remedy compared with vaccine treatment—well, it is incomparable."

Professor Wm. H. Dieffenbach of the New York Medical College and Hospital for Women states: "I endorse Dr. Duncan's method autotherapy as strong as pen and ink can make it."

Autotherapy is endorsed in the highest terms by the deans of seven large medical colleges in the United States. The Veterinary Society of the County of New York after putting autotherapy to many crucial tests on animals and reviewing the reports of veterinary army surgeons endorse autotherapy as strong as the English language can make it, and elected the discoverer and founder an honorary member of their organization. Thousands of similar endorsements could be given from independent physicians and surgeons scattered in all parts of the earth. It may not be out of place to give one other endorsement, as it may lead some who are not of the regular school of medicine to adopt it, in the treatment of wounds incident to the mobilization of our troops and to those of warfare, if we should be unfortunate to be so entangled.

The Homeopathic Medical Society of the county of New York at one of its regular monthly meetings, after an official investigation extending over a year, unanimously endorsed autotherapy with all honor and praise.

Autotherapy in its application to the prevention and cure of purulent infection, both in and out of the army, speaks for itself incontrovertibly in results: it needs no defense and but little explanation. The principle upon which cures made by its use rests is in accord with the modern conception of biological therapeutics. The technic is superlatively simple: the results absolutely dependable. This method is no longer an experiment, it has come to the physician's hand to stay, for the principle underlying the cures made by its use is everlasting and immutable. A few drops of pus will cure a purulent infection a thousand years from now as well as it does today.

The more virulent the infecting micro-organisms (both causative and complicating) the quicker will be the response and cure.

The technic of the oral administration of pus by the mouth in infected wounds needs no elaboration at this time, as so much has previously been written on the subject, but in order that the army surgeons at the front may be thoroughly prepared to meet most successfully these conditions, it has been deemed advisable to give at this time a convenient working formula.

The dog in licking his wounds gives himself a dose of the unmodified autogenous toxins, or the autotherapeutic remedy; for this reason his wounds heal quickly; he never has a bad infection except on the head, where for anatomical reasons he cannot lick.

Placing pyogenic organisms in the mouth early raises the power of the blood serum and stimulates the activity of the leucocytes quickly to overcome the invaders. Too often we hear of physicians and surgeons infecting their hands during an operation or autopsy and dying from sepsis. If the surgeon will remember to suck the wound then and there and afterwards when there is any irritation in it there will be no more deaths from this source, for the wound will heal by first intention. Too great publicity in the army cannot be given to this simple therapeutic measure; homely it may appear but in therapeutic value it surpasses anything that modern medicine or surgery has given us for this condition.

In punctured and gunshot wounds in which foreign material such as cloth, wood, etc., has been driven into the tissues, remove the foreign body before antiseptics are applied and place it in the patient's mouth, instructing him to chew it for five minutes, swallowing the fluid, spitting out the material, then here will in all probability be placed in the mouth some of the micro-organisms that entered the wound. We know when this occurs there will be a probability almost amounting to a certainty that infection will be prevented. The wounded soldier should be thoroughly instructed to lick his wounds as soon as they are received and then many times during the day, or when there is irritation in the parts. If they do this, purulent infection will be aborted. If the wound is so situated where from anatomical reasons he cannot lick it, the wounded soldier should be instructed to place over the wound a small clean piece of gauze which he carries by army regulations, and to replace it every twelve hours, and to chew the blood-stained portions of the gauze for five minutes and swallow the fluids. If he does this properly, purulent infection will be aborted.

The above is applicable to the largest class of wounds of warfare; that is, those that are in no way connected with the alimentary tract or respiratory system. By this elementary simple means *the wounded soldier has it within his power to abort infection often before he is seen by the surgeon.* The importance of this cannot be overestimated. This is particularly applicable to wounds of the brain that so often otherwise have a fatal termination. The treatment should be continued twice daily for at least ten days.

It is just as important that the wounded soldier should be instructed to treat similarly many minor infections that are incident to a Northern man being thrust into the hot sweltering climate of Southern Texas and Northern Mexico, namely, as small suppurations following the thrust from the needles of the chincapin, chaparral, Spanish bayonet, cactus, etc., or the suppuration following the head of a tick that has burrowed into the tissues, or the bite from any other insects which infest that region.

The resistance of his tissues is reduced by the hot sweltering days and his inability to sleep at night from the pain incident to the inflammatory condition of his skin resulting from the red bugs, ferocious mosquitoes, other insect bites, thorns, etc.

Whenever there are five drops of pus or less in any of these wounds, the soldier should be instructed to lick the wound or take the pus from it in his mouth; for in this way, and this way only, will he be able to *treat himself* in the quickest, cheapest, most convenient and best manner possible. By so doing he'll not only be able to cure these infections quickly and prevent much suffering, but he will preserve his vitality and immunize himself against pyogenic micro-organisms, so that if he should be seriously wounded he would be more liable to resist infection than he would be otherwise.

Wounds of the alimentary tract and respiratory system, including wounds of the nose, mouth, larynx, esophagus, etc., should be treated by the hypodermic injection method, if infection is to be aborted or cured quickly. It will be remembered that practically all accidental wounds are infected and even in operative wounds the sterilization of the skin is never absolute. Autotherapy presupposes the wound is infected and utilizes the toxic products of the micro-organisms or the micro-organisms themselves to combat the infection.

The writer was the first to successfully employ the live pathogenic micro-organisms as a therapeutic agent.

A fresh wound usually bleeds to some extent, the pyogenic micro-organisms in the wound will tend to be washed out with the blood. Some of the micro-organisms are probably destroyed by the blood plasma; those remaining in the wound that proliferate and those that come out with the bleeding engage our attention from an autotherapeutic point of view. Stress has always been laid on the fact that vaccines should always be given early to be most effective, but the unmodified toxin complex is extremely efficacious at any stage of the infection. With autotherapy we may practically throw antiseptic and aseptic surgery to the winds and never have an apparent infection follow wounds of warfare.

The abortion of infections in the intra-alimentary and intrapulmonary wounds is similar in technic to the cure of infections in these regions, except that in the abortion of infections the hypodermic injections of the filtrate may be given once a day, whereas in the cure of purulent infection it is given as will be pointed out in a later paragraph only according to the needs of the patient.

In wounds that close easily a small gauze drain should be inserted to keep it open, then it may be employed autotherapeutically; i. e., by chewing it for five minutes, swallowing the fluids and spitting out the drain or by the filtration method described later. The efficacy of the drain is therefore twofold, first, collecting the micro-organisms on it in order that they may be employed autotherapeutically; second, allowing the exudate to come to the surface. It is conceded of course that shock, loss of blood, churning of the tissues, foreign bodies in the wound, etc., complicate the conditions, and the good effect of fortifying the tissues to the infecting micro-organisms under these conditions by autotherapeutic immunization might not be apparent as under conditions we should ordinarily expect to find in a wound. It is assumed that the surgeon will remove all foreign bodies and treat the wound otherwise by well known surgical procedures. In this discussion we are dealing with wounds uncomplicated except by pyogenic micro-organism. However, in all wounds this treatment tends to be beneficial, for under any condition in which we could obtain the infecting micro-organisms we should be able to fortify against their invasion sooner and better than any other method heretofore advanced.

In treating wounds autoseptically, I wish distinctly to be understood as neither advocating nor recommending any extraneous material or foreign micro-organisms be placed in the wound, either by careless or wilful handling of the wound, although in my test cases I have handled many accidental wounds that appeared to be unsterile, with unsterile fingers and instruments, and I have invariably had my test cases heal without apparent infection. Aseptic technic should be employed as far as possible; *autoseptic* technic should be employed also as an additional element of safety. For if aseptic surgical technic has for any reason been faulty; or, if for reasons little understood at the present time, the wound becomes septic, autosepsis will then tend to build up the bacterial element of the blood to the infecting micro-organism. In this way the wound is made to heal without infection being apparent. We thus put out the match, as it were, before the conflagration is well started.

(To be continued in September issue.)

